# Risks of Restrictive Versus Liberal Red Blood Cell Transfusion Strategies in Patients With Cardiovascular Disease: An Updated Meta-Analysis

**DOI:** 10.1161/CIRCOUTCOMES.124.010957

**Published:** 2024-05-15

**Authors:** Willard N. Applefeld, Verity J. Ford, Irene Cortes-Puch, Jeffrey Wang, Junfeng Sun, Tracy C. Shields, Robert L. Danner, Peter Q. Eichacker, Michael A. Solomon, Harvey G. Klein, Charles Natanson

**Affiliations:** 1 Division of Cardiology, Duke University Medical Center, Durham, NC (W.N.A.).; 2 Critical Care Medicine Department, Clinical Center (V.J.F., J.S., R.L.D., P.Q.E., M.A.S., C.N.), National Institutes of Health, Bethesda, MD.; 3 NIH Library, Office of Research Services, Office of the Director (T.C.S.), National Institutes of Health, Bethesda, MD.; 4 Department of Transfusion Medicine, Clinical Center (H.G.K.), National Institutes of Health, Bethesda, MD.; 5 National Heart, Lung and Blood Institute (R.L.D., M.A.S., C.N.), National Institutes of Health, Bethesda, MD.; 6 Division of Pulmonary, Critical Care and Sleep Medicine, UC Davis Medical Center, Sacramento, CA (I.C.-P.).; 7 Division of Cardiology, Emory University School of Medicine, Atlanta, GA (J.W.).

**Keywords:** anemia, cardiac surgical procedures, erythrocytes, orthopedic procedures, uncertainty

In patients with acute coronary syndromes (ACSs), anemia has long been recognized to be an independent predictor of major adverse cardiovascular events.^1^ The 2023 Association for Advancement of Blood and Biotherapies international guidelines state that “for patients with acute and chronic ischemic cardiac disease, there remains substantial uncertainty regarding the safety of restrictive red blood cell (RBC) transfusion thresholds” but continue to recommend a threshold of “8 g/dL for patients undergoing orthopedic surgery or those with preexisting cardiovascular disease” (CVD).^2^ The guidance relies on a series of randomized transfusion trigger trials (RTTTs) comparing liberal versus restrictive red blood cell transfusion thresholds.^2,3^ Seminal RTTTs, in their original publications, did not delineate a number of important outcomes in subjects with CVD. The absence of data about cardiac outcomes of interest dates to one of the first RTTTs published in 1999 (see the Figure and Appendix A in the Data Supplement for all RTTT references included in this meta-analysis). In a secondary analysis,^4^ we found after separating out key subgroups that restrictive and liberal triggers had significantly different and opposite effects on mortality. In that 1999 trial, the restrictive trigger increased mortality in subjects with CVD, whereas the liberal trigger increased mortality in subjects without CVD.^3,4^ In 2011, the largest and arguably most influential RTTT (Figure) did not report the outcome of ACS in subjects with CVD among the ≈2000 orthopedic surgery patients enrolled. We obtained these data from the authors and reported this outcome publicly for the first time 7 years later, in our 2018 meta-analysis.^3^ In the 2011 orthopedic trial, subjects with CVD were significantly more likely to develop ACS in the restrictive compared with the liberal arm.^3^ In our 2018 meta-analysis, which analyzed trials cumulatively enrolling > 5000 subjects with and without CVD, we found that those treated in restrictive arms had overall also a significantly increased risk of the outcome of ACS.^3^ We now update our meta-analysis^3^ to include any data available from subjects with CVD enrolled in RTTT since 2018.

**Figure.**
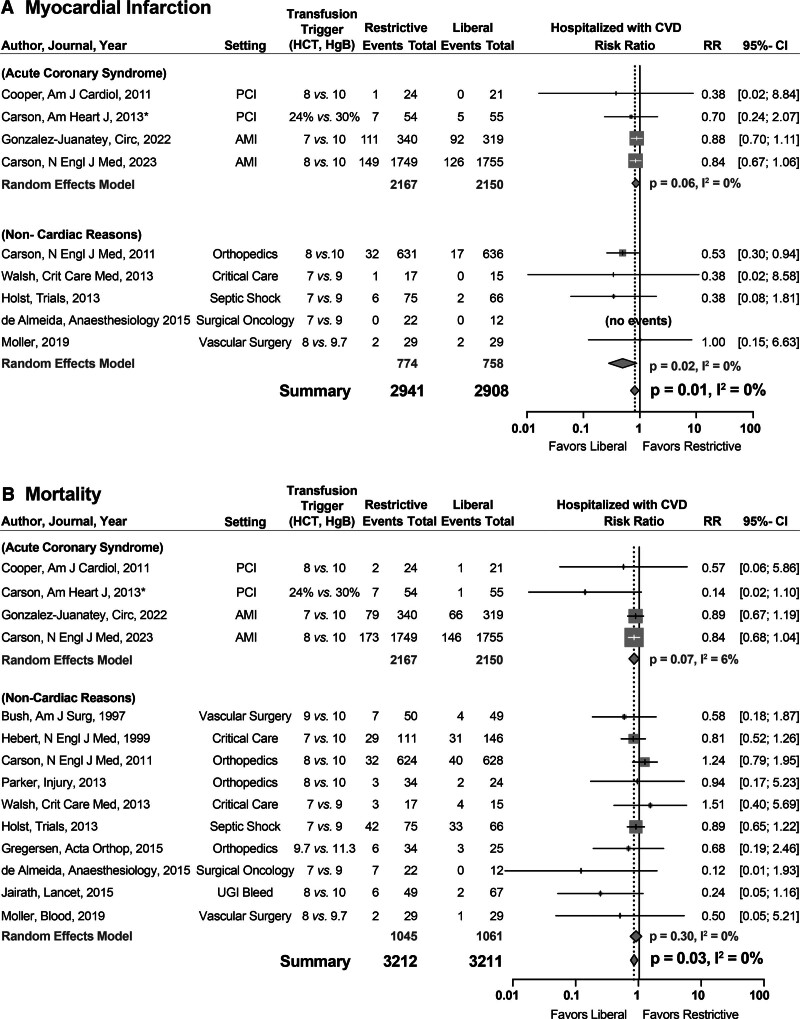
**The relative risks in hospitalized individuals with pre-existing cardiovascular disease receiving a liberal versus restrictive transfusion trigger.** Relative risk of a myocardial infarction (**A**) and death (**B**) in individuals receiving a liberal vs restrictive transfusion trigger not undergoing cardiac surgery with preexisting cardiovascular disease (CVD) and hospitalized for acute coronary syndrome (top of each part) or noncardiac reasons (bottom of each part). The study by Carson in 2013* published in the *American Heart Journal* is found listed under “Subjects With Acute Coronary Syndrome.” However, some of these subjects may have been undergoing percutaneous coronary intervention (PCI) for stable angina. For full citation of papers listed in the first column, please see Appendix A in the Data Supplement. This meta-analysis analytic plan was prospectively registered in PROSPERO (ID 485084) before conducting any searches. Risk ratios (RRs) of mortality and acute coronary syndrome were analyzed using random-effects models (Junfeng Sun), with 0.5 added to each cell in studies with zero cells. Heterogeneity among studies was assessed using the Q statistic and I^2^ value. All analyses were performed using R, version 4.3.1, with package meta, version 6.5-0. AMI indicates acute myocardial infarction; HCT, hematocrit; HgB, hemoglobin; and UGI, upper gastrointestinal. *Some subjects enrolled had stable angina.

The purpose of this meta-analysis was to find additional RTTTs since our 2018 meta-analysis that enrolled subjects with CVD hospitalized for ACS or noncardiac indications comparing liberal versus restrictive transfusion trigger strategy and reported the outcomes of mortality or additional ACS.^3^ The largest, most influential cardiac surgery RTTT had a highly significant qualitative interaction in which younger subjects benefited from, whereas older subjects did worse with, a liberal transfusion trigger.^5^ Because we could not adequately account for such an interaction in our meta-analysis, we prospectively excluded cardiothoracic surgery trials. We used the most up-to-date trial data if there was >1 RTTT publication. The information in this meta-analysis is publicly available; thus, internal review board approval was not needed.

Our search (Tracey Shields) on November 23, 2023, using the same terms, data bases, and strategy used in 2018 yielded 997 additional studies published from 2018 to 2023, 3 were unique RTTTs (Figure) enrolling 4222 subjects that met inclusion criteria. This brought the number of noncardiac surgery RTTTs enrolling subjects with CVD hospitalized for ACS or for other reasons analyzed to 14. Of 3211 subjects with CVD with mortality data in liberal arms, 334 died (10.4% mortality). Of 3212 subjects with CVD with mortality data in the restrictive arms, 398 died (12.4% mortality). The subjects in the liberal arms had significantly lower risk of death compared with the restrictive arms (relative risk of death, 0.87 [95% CI, 0.76–0.99]; *P*=0.03; I^2^=0%; Figure). Of 2908 subjects with CVD with available data in liberal arms, 244 experienced ACS (8.4%); of 2941 subjects with CVD with available data in the restrictive arms, 309 experienced ACS (10.5%). The subjects in the liberal arms had significantly lower risk of ACS compared with the restrictive arms (relative risk of death, 0.82 [95% CI, 0.70–0.96]; *P*=0.01; I^2^=0%).

In individuals with preexisting CVD who are hospitalized for ACS, orthopedic surgery, or other noncardiac surgery indications, the restrictive transfusion triggers (as shown in the Figure; range, 7–9.7; median, 8 g/dL) were associated with a significant increase in the outcomes of ACS and mortality rates. The most recent and largest RTTT studying subjects with acute myocardial infarctions used a restrictive hemoglobin threshold of 7 or 8 g/dL and achieved on average a hemoglobin of 8.9 g/dL in the restrictive arm. The restrictive trigger threshold was associated with an increased combined risk of myocardial infarction and death as compared with hemoglobin of 10.4 g/dL in the liberal threshold arm (*P*=0.07; Figure). This is consistent with the findings of our meta-analysis, which has many more subjects enrolled, giving our study the added statistical power needed to show significance. We conclude that the summary data support the use of a liberal transfusion threshold as opposed to a restrictive approach for patients with CVD. Physicians must consider pertinent cardiac comorbidities and other clinical variables when making decisions about appropriate liberal transfusion thresholds. Whereas such a statement is usually appended to transfusion guidelines, too often the hemoglobin value alone remains the trigger for transfusion. Transfusion practice guidelines should update recommendations to incorporate the mounting evidence for harm when restrictive thresholds are used for patients with CVD with ACS or hospitalized for noncardiac reasons.

## ARTICLE INFORMATION

### Sources of Funding

This study was supported by the National Institutes of Health (NIH) intramural funding from the NIH Clinical Center. The work by the authors was conducted as part of the US Government–funded research; however, the opinions expressed are not necessarily those of the NIH.

### Disclosures

None.

### Supplemental Material

Appendix A: Full Citations of Studies Included in Meta-analysis

PRISMA Checklist

## Supplementary Material
