# Supplementary material for: Risks of Restrictive Versus Liberal Red Blood Cell Transfusion Strategies in Patients With Cardiovascular Disease: An Updated Meta-Analysis
Source: Circ Cardiovasc Qual Outcomes. 2024 May 15;17(6):e010957. doi: 10.1161/CIRCOUTCOMES.124.010957 (PMC11186712; doi:10.1161/CIRCOUTCOMES.124.010957)
Supplement: Supplementary file 2 [file hcq-17-e010957-s002.pdf]

Risks of Restrictive Versus Liberal Red Blood Cell Transfusion Strategies in Patients with Cardiovascular Disease: An Updated Meta-analysis

Willard N. Applefeld MD, Verity Ford MD, Irene Cortes-Puch MD, Jeffrey Wang MD, Junfeng Sun PhD, Tracy C. Shields MSIS, Robert L. Danner MD, Peter Q. Eichacker MD, Michael A. Solomon MD MBA, Harvey G. Klein MD, and Charles Natanson MD

Appendix A: Full Citations of Studies Included in Meta-analysis

Myocardial infarction

Cooper HA, Rao SV, Greenberg MD, Rumsey MP, McKenzie M, Alcorn KW, Panza JA. Conservative versus liberal red cell transfusion in acute myocardial infarction (the CRIT Randomized Pilot Study). *Am J Cardiol*. 2011;108(8):1108-1111. doi: 10.1016/j.amjcard.2011.06.014

Carson JL, Brooks MM, Abbott JD, Chaitman B, Kelsey SF, Triulzi DJ, Srinivas V, Menegus MA, Marroquin OC, Rao SV, Noveck H, Passano E, Hardison RM, Smitherman T, Vagaonescu T, Wimmer NJ, Williams DO. Liberal versus restrictive transfusion thresholds for patients with symptomatic coronary artery disease. *Am Heart J*. 2013;165(6):964-971.e1. doi: 10.1016/j.ahj.2013.03.001.

Gonzalez-Juanatey JR, Lemesle G, Puymirat E, Ducrocq G, Cachanado M, Arnaiz JA, Martínez-Sellés M, Silvain J, Ariza-Solé A, Ferrari E, Calvo G, Danchin N, Avendano-Solá C, Rousseau A, Vicaut E, Gonzalez-Ferrero T, Steg PG, Simon T, REALITY Investigators. One-Year Major Cardiovascular Events After Restrictive Versus Liberal Blood Transfusion Strategy in Patients With Acute Myocardial Infarction and Anemia: The REALITY Randomized Trial. *Circulation*. 2022;145(8)486-488. doi: 10.1161/CIRCULATIONAHA.121.057909.

Carson JL, Brooks MM, Hébert PC, Goodman SG, Bertolet M, Glynn SA, Chaitman BR, Simon T, Lopes RD, Goldsweig AM, DeFilippis AP, Abbott JD, Potter BJ, Carrier FM, Rao SV, Cooper HA, Ghafghazi S, Fergusson DA, Kostis WJ, Noveck H, Kim S, Tessalee M, Ducrocq G, de Barros E Silva PGM, Triulzi DJ, Alsweiler C, Menegus MA, Neary JD, Ulh L, Strom JB, Fordyce CB, Ferrari E, Silvain J, Wood FO, Daneault B, Polonsky TS, Senaratne M, Puymirat E, Bouleti C, Lattuca B, White HD, Steg PG, Alexander JH, MINT Investigators. Restrictive or Liberal Transfusion Strategy in Myocardial Infarction and Anemia. *N Engl J Med*. 2023;389(26):2446-2456. doi: 10.1056/NEJMoa2307983.

Carson JL, Terrin ML, Noveck H, Sanders DW, Chaitman BR, Rhoads GG, Nemo G, Dragert K, Beaupre L, Hildebrand K, Macaulay W, Lewis C, Cook DR, Dobbin G, Zakriya JK, Apple FS, Horney RA, Magaziner J, FOCUS Investigators. Liberal or restrictive transfusion in high-risk patients after hip surgery. *N Engl J Med*. 2011;365(26):2453-2462. doi: 10.1056/NEJMoa1012452.

Walsh TS, Boyd JA, Watson D, Hope D, Lewis S, Krishan A, Forbes JF, Ramsay P, Pearse R, Wallis C, Cairns C, Cole S, Wyncoll D, RELIEVE Investigators. Restrictive versus liberal transfusion strategies for older mechanically ventilated critically ill patients: a randomized pilot trial. *Crit Care Med*. 2013;41(10):2354-2363. doi: 10.1097/CCM.0b013e318291cce4.

Holst LB, Haase N, Wetterslev J, Wernerman J, Aneman A, Guttormsen AB, Johansson PI, Karlsson S, Klemenzson G, Winding R, Nebrich L, Albeck C, Vang ML, Bülow HH, Elkjær JM, Nielsen JS, Kirkegaard P, Nibro H, Lindhardt A, Strange D, Thormar K, Poulsen LM, Berezowicz P, Bådstøløkken PM, Strand K, Cronhjort M, Haunstrup E, Rian O, Oldner A, Bendtsen A, Iversen S, Langva JA, Johansen RB, Nielsen N, Pettilä V, Reinikainen M, Keld D, Leivdal S, Breider JM, Tjäder I, Reiter N, Gøttrup U, White J, Wiis J, Andersen LH, Steensen M, Pernerde A. Transfusion requirements in septic shock (TRISS) trial - comparing the effects and safety of liberal versus restrictive red blood cell transfusion in septic shock patients in the ICU: protocol for a randomised controlled trial. *Trials*. 2013;14:150. doi: 10.1186/1745-6215-14-150.

de Almeida JP, Vincent JL, Galas FR, de Almeida EP, Fukushima JT, Osawa EA, Bergamin F, Park CL, Nakamura RE, Fonseca SM, Cutait G, Alves JI, Bazan M, Vieira S, Sandrini AC, Palomba H, Ribeiro U Jr, Crippa A, Dalloglio M, Diz Mdel P, Kalil Filho R, Auler JO Jr, Rhodes A, Hajjar LA. Transfusion requirements in surgical oncology patients: a prospective, randomized controlled trial. *Anesthesiology*. 2015;122(1):29-38. doi: 10.1097/ALN.0000000000000511.

Møller A, Nielsen HB, Wetterslev J, Pedersen OB, Hellemann D, Winkel P, Marcussen KV, Ramsing BGU, Mortensen A, Jakobsen JC, Shahidi S. Low vs high hemoglobin trigger for transfusion in vascular surgery: a randomized clinical feasibility trial. *Blood*. 2019;133(25):2639-2650. doi: 10.1182/blood-2018-10-877530.

Myocardial infarction

Cooper HA, Rao SV, Greenberg MD, Rumsey MP, McKenzie M, Alcorn KW, Panza JA. Conservative versus liberal red cell transfusion in acute myocardial infarction (the CRIT Randomized Pilot Study). *Am J Cardiol.* 2011;108(8):1108-1111. doi: 10.1016/j.amjcard.2011.06.014

Carson JL, Brooks MM, Abbott JD, Chaitman B, Kelsey SF, Triulzi DJ, Srinivas V, Menegus MA, Marroquin OC, Rao SV, Noveck H, Passano E, Hardison RM, Smitherman T, Vagaonescu T, Wimmer NJ, Williams DO. Liberal versus restrictive transfusion thresholds for patients with symptomatic coronary artery disease. *Am Heart J*. 2013;165(6):964-971.e1. doi: 10.1016/j.ahj.2013.03.001.

Gonzalez-Juanatey JR, Lemesle G, Puymirat E, Ducrocq G, Cachanado M, Arnaiz JA, Martínez-Sellés M, Silvain J, Ariza-Solé A, Ferrari E, Calvo G, Danchin N, Avendano-Solá C, Rousseau A, Vicaut E, Gonzalez-Ferrero T, Steg PG, Simon T, REALITY Investigators. One-Year Major Cardiovascular Events After Restrictive Versus Liberal Blood Transfusion Strategy in Patients With Acute Myocardial Infarction and Anemia: The REALITY Randomized Trial. *Circulation*. 2022;145(8)486-488. doi: 10.1161/CIRCULATIONAHA.121.057909.

Carson JL, Brooks MM, Hébert PC, Goodman SG, Bertolet M, Glynn SA, Chaitman BR, Simon T, Lopes RD, Goldsweig AM, DeFilippis AP, Abbott JD, Potter BJ, Carrier FM, Rao SV, Cooper HA, Ghafghazi S, Fergusson DA, Kostis WJ, Noveck H, Kim S, Tessalee M, Ducrocq G, de Barros E Silva PGM, Triulzi DJ, Alsweiler C, Menegus MA, Neary JD, Ulh L, Strom JB, Fordyce CB, Ferrari E, Silvain J, Wood FO, Daneault B, Polonsky TS, Senaratne M, Puymirat E, Bouleti C, Lattuca B, White HD, Steg PG, Alexander JH, MINT Investigators. Restrictive or Liberal Transfusion Strategy in Myocardial Infarction and Anemia. *N Engl J Med*. 2023;389(26):2446-2456. doi: 10.1056/NEJMoa2307983.

Bush RL, Pevec WC, Holcroft JW. A prospective, randomized trial limiting perioperative red blood cell transfusions in vascular patients. *Am J Surg*. 1997;174(2):143-148. doi: 10.1016/s0002-9610(97)00073-1.

Hébert PC, Wells G, Blajchman MA, Marshall J, Martin C, Pagliarello G, Tweeddale M, Schweitzer I, Yetisir E. A multicenter, randomized, controlled clinical trial of transfusion requirements in critical care. Transfusion Requirements in Critical Care Investigators, Canadian Critical Care Trials Group. *N Engl J Med*. 1999;340(6):409-4017. doi: 10.1056/NEJM199902113400601.

Carson JL, Terrin ML, Noveck H, Sanders DW, Chaitman BR, Rhoads GG, Nemo G, Dragert K, Beaupre L, Hildebrand K, Macaulay W, Lewis C, Cook DR, Dobbin G, Zakriya JK, Apple FS, Horney RA, Magaziner J, FOCUS Investigators. Liberal or restrictive transfusion in high-risk patients after hip surgery. N Engl J Med. 2011;365(26):2453-2462. doi: 10.1056/NEJMoa1012452.

Parker MJ. Randomised trial of blood transfusion versus a restrictive transfusion policy after hip fracture surgery. *Injury*. 2013;44(12):1916-1918. doi: 10.1016/j.injury.2013.04.033.

Walsh TS, Boyd JA, Watson D, Hope D, Lewis S, Krishan A, Forbes JF, Ramsay P, Pearse R, Wallis C, Cairns C, Cole S, Wyncoll D, RELIEVE Investigators. Restrictive versus liberal transfusion strategies for older mechanically ventilated critically ill patients: a randomized pilot trial. Crit Care Med. 2013;41(10):2354-2363. doi: 10.1097/CCM.0b013e318291cce4.

Holst LB, Haase N, Wetterslev J, Wernerman J, Aneman A, Guttormsen AB, Johansson PI, Karlsson S, Klemenzson G, Winding R, Nebrich L, Albeck C, Vang ML, Bülow HH, Elkjær JM, Nielsen JS, Kirkegaard P, Nibro H, Lindhardt A, Strange D, Thormar K, Poulsen LM, Berezowicz P, Bådstøløkken PM, Strand K, Cronhjort M, Haunstrup E, Rian O, Oldner A, Bendtsen A, Iversen S, Langva JA, Johansen RB, Nielsen N, Pettilä V, Reinikainen M, Keld D, Leivdal S, Breider JM, Tjäder I, Reiter N, Gøttrup U, White J, Wiis J, Andersen LH, Steensen M, Pernerde A. Transfusion requirements in septic shock (TRISS) trial - comparing the effects and safety of liberal versus restrictive red blood cell transfusion in septic shock patients in the ICU: protocol for a randomised controlled trial. *Trials*. 2013;14:150. doi: 10.1186/1745-6215-14-150.

Gregersen M, Borris LC, Damsgaard EM. Postoperative blood transfusion strategy in frail, anemic elderly patients with hip fracture: the TRIFE randomized controlled trial. *Acta Orthop*. 2015;86(3):363-372. doi: 10.3109/17453674.2015.1006980.

de Almeida JP, Vincent JL, Galas FR, de Almeida EP, Fukushima JT, Osawa EA, Bergamin F, Park CL, Nakamura RE, Fonseca SM, Cutait G, Alves JI, Bazan M, Vieira S, Sandrini AC, Palomba H, Ribeiro U Jr, Crippa A, Dalloglio M, Diz Mdel P, Kalil Filho R, Auler JO Jr, Rhodes A, Hajjar LA. Transfusion requirements in surgical oncology patients: a prospective, randomized controlled trial. Anesthesiology. 2015;122(1):29-38. doi: 10.1097/ALN.0000000000000511. Jairath, Lancet, 2015

Jairath V, Kahan BC, Gray A, Doré CJ, Mora A, James MW, Stanley AJ, Everett SM, Bailey AA, Dallal H, Greenaway J, Le Jeune I, Darwent M, Church N, Reckless I, Hodge R, Dyer C, Meredith S, Llewelyn C, Palmer KR, Logan RF, Travis SP, Walsh TS, Murphy MF. Restrictive versus liberal blood transfusion for acute upper gastrointestinal bleeding (TRIGGER): a pragmatic, open-label, cluster randomised feasibility trial. *Lancet*. 2015;386(9989):137-144. doi: 10.1016/S0140-6736(14)61999-1.

Møller A, Nielsen HB, Wetterslev J, Pedersen OB, Hellemann D, Winkel P, Marcussen KV, Ramsing BGU, Mortensen A, Jakobsen JC, Shahidi S. Low vs high hemoglobin trigger for transfusion in vascular surgery: a randomized clinical feasibility trial. Blood. 2019;133(25):2639-2650. doi: 10.1182/blood-2018-10-877530.
